# A randomized controlled trial of a self-led, virtual reality-based cognitive behavioral therapy on sick role adaptation in colorectal cancer patients: study protocol

**DOI:** 10.1186/s12885-024-12583-1

**Published:** 2024-07-17

**Authors:** Xinxin Li, Xiaodan Wu, Chao Chen, Huaxiang Chao, Jingyue Xie, Qianqian Du, Meifen Zhang

**Affiliations:** 1 School of Nursing, Sun Yat-Sen University, Guangzhou, 510080 China; 2 State Key Laboratory of Oncology in South China, Collaborative Innovation Center for Cancer Medicine, Sun Yat-sen University Cancer Center, Guangzhou, 510060 China; 3 School of Computer Science and Engineering, Sun Yat-Sen University, Guangzhou, 510006 China

**Keywords:** Colorectal cancer, Virtual reality, Cognitive behavioral therapy, Role adaptation, Randomized controlled trial, Study protocol

## Abstract

**Background:**

Significant concomitants of the sick role maladaptation in colorectal cancer (CRC) patients include inappropriate cognitions, emotional states, and overt conducts associated to disease. This protocol was developed to implement and evaluate the effects of a self-led, virtual reality-based cognitive behavioral therapy (VR-CBT) on the sick role adaptation among working-age CRC patients.

**Methods:**

This is an assessor-blinded, randomized controlled trail that adheres to the SPIRIT 2013 Statement guidelines. A total of 60 working-age CRC patients will be recruited from the colorectal wards of a cancer center and randomly assigned to the VR-CBT group or attention control (AC) group. The VR-CBT group will receive a 7-sessions VR-CBT targeted to sick role adaptation, while the AC group will receive weekly attention at the same time the VR-CBT group receives the intervention. The sick role adaptation, anxiety and depression, illness perceptions, and quality of life will be measured at baseline, 1, 2 and 3-month after completion of the intervention. Side-effects related to VR in the VR-CBT group will be measured at the end of each session. The participants will receive invitations to participate in semi-structured interviews to explore their experiences with the intervention.

**Discussion:**

The positive outcomes and user experience of VR-CBT will advance researches on the effectiveness of psychosocial interventions that aims to promote adaptation to the unexpected sick role on cancer populations. This protocol can be tested as an accessible and feasible alternative to traditional high-cost treatment in a randomized controlled study to improve the outcomes of younger cancer survivors.

Trial registration.

The protocol was registered on 21 June, 2023 in Chinese Clinical Trial Registry (No.: ChiCTR2300072699) at https://www.chictr.org.cn/.

## Background

Globally, colorectal cancer (CRC) is the third most commonly diagnosed cancer and the second major cause of cancer death in both sexes [1]. In 2020, newly diagnosed CRC patients in China occupied 28.8% of all new cases worldwide [1]. Although CRC typically affects older populations, the onset age of the disease has been getting younger [2]. A body of international literature reveals that there has been an observed rise in the prevalence of CRC among younger adults under the age of 50 of both sexes, constituting approximately 10% of the total new cases of CRC reported [2, 3]. Poorer differentiation, more advanced stage, and worse prognosis are typically witnessed in younger patients with CRC [3]. Further, they may experience psychosocial impairment due to bowel dysfunction, urinary problems, sexual dysfunction, and the possibility of an ostomy following rectal resection[4].

The prevalence of CRC among the younger population sparked a concern that CRC patients at working age would confront an undesirable role—being young and sick—that conflicted with their stage of life and their crucial roles as the backbone of their nuclear and extended family [5, 6]. It is reported that people diagnosed during their working years have lower quality of life (QOL), more practical problems, and worse psychosocial consequences than older-onset CRC patients aged over 60 [2, 7]. This might be as a result of the numerous difficulties working-age CRC patients face, who are managing multiple roles at the same time as taking on the role of a cancer patient at an unexpectedly young age, suffering more detrimental and maladaptive psychosocial effects than their older counterparts [7]. It has been demonstrated that cancer-related identities are associated with adjustment, particularly psychological well-being [8].

In the sociological literature, illness refers to an incapacity of an individual to effectively perform social roles [9]. Talcott Parsons [10] emphasized that the sociocultural context in which disease occurs allows for the definition of a particular social role—the sick role—that people may occupy during a period of illness. The sick role is the core patient role within the medical system and is considered an irregular or dysfunctional societal role [11, 12]. Suffering, helplessness, disability, and the risk, or even certainty of death following diagnosis and treatment represent profound disruptions to the prior role expectations [10]. It is therefore challenging for patients to adjust to the unexpected sick role, especially for those in their young and middle adulthood with limited illness experience and less effective coping strategies first encountered with surgery. The sick role adaptation refers to regulation of self-identity, accommodation of cognitive and behavioral responses following the diagnosis [13]. In other words, inappropriate cognitions, emotional states, and overt conducts associated to disease are all important concomitants of the sick role maladaptation [14].

Commonly reported experiences of CRC patients during the preoperative stage include fear, helplessness, isolation, devastation and uncertainty [15, 16]. Postoperatively, CRC patients are confronted with multifaceted psychological challenges include severe anxiety and depression, embarrassment, loss of dignity, as well as worse social and emotional role functioning till 4–6 weeks after surgery [16–19]. To deal with these stressful and traumatic challenges, patients with CRC need to make major psychosocial adaptations [20]. Therefore, it is noted that technology-based interventions are proposed to deliver preoperatively to facilitate CRC patients to accept their diagnosis and adjust to life following surgery [16]. Patients must incorporate their cancer experience into their self-concept required by the uncertainty and chronicity of cancer. This may be especially essential for people who are diagnosed with cancer while they are still in their working age because they must complete normal developmental tasks such as identity formation, intimate relationships, and financial independence in addition to managing their cancer treatments [8]. However, to date, previous studies focused on psychosocial interventions for CRC patients have not dealt with the issue of sick role adaptation.

A literature review showed that cognitive behavioral therapy (CBT) was one of the most common and effective intervention in improving QOL and alleviating adverse psychological effects among CRC survivors [20]. One explanation could be that CBT interventions for patients cover a wide variety of aspects, including social, psychological, and physical components, which can enhance QOL and alleviate negative psychological impacts [21]. However, the requirement for experienced practitioners and the time as well as the resources required to prepare one, hinders the widespread adoption of CBT in general practice [22]. With the growing proliferation of smartphones, CBT delivered by mobile application (app) becomes an attractive option since it is cost-effective and highly accessible. Interventions built upon CBT principles and delivered via a mobile app has been shown to be as effective as face-to-face CBT with stronger benefits in cancer patients [22, 23].

The development of virtual reality (VR) technology provides full control over the user experience, which can be used therapeutically to alleviate some of the negative effects of illness by enabling people to "escape" from their real world to pleasant environments where they can have more positive thoughts and feelings [24, 25]. This tool has shown several advantages that are noteworthy for optimizing psychotherapies including its versatility, acceptability as well as adherence to treatment [26]. Because of these advantages, VR-based therapy has been developed and applied to evaluating and treating various psychological issues [27]. The majority of previous studies on VR-based therapies for cancer patients have focused on reducing anxiety, fatigue and pain [28–30]. For many existing CBT procedures, VR has been regarded a therapeutically appropriate treatment modality, and for its more experiential component, VR-based approaches may increase the possibility of transferring knowledge and skills gained during VR sessions to patients’ everyday life [25, 26]. Since VR can be used frequently, delivering CBT techniques such as breath relaxation with VR can enhance its effectiveness [31]. There is ample evidence to support efficacy of VR-based interventions in psychotherapy and cancer populations with improved emotional, cognitive, and physical well-being [32, 33], there is, however, a striking lack of VR-based CBT therapies for CRC patients. As a result, it is necessary to develop a self-led VR-based CBT intervention on the sick role adaptation among working-age CRC patients and explore its effectiveness.

**Fig. 1:**
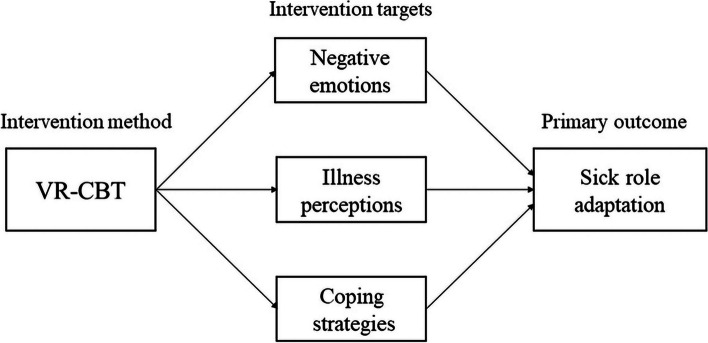
The causal model of this study

The Common Sense Model of Self-regulation (CSM) is probably the most prevalent theory to explain and predict how patient adapt to their disease and behave [34]. According to CSM, an understanding of adaptability to health threats originates in an analysis of threat from the lay perspective of the individual, in particular, is essential for initiating subsequent coping strategies aimed at both threat and emotion management. These two simultaneous processes constitute a self-regulatory system with substantial consequences for illness outcomes such as adaptation to illness [35]. Previously, the combination of CSM and CBT technique has been applied in patients with chronic disease [34]. CBT is therefore proposed as a potentially beneficial approach to improve the sick role adaptation among CRC patients by reconstructing their beliefs of CRC, adjusting negative emotions and adopting effective coping strategies. Based on CSM and CBT technique, emotion, cognition, and coping were the basis for the development of the intervention strategies in our study, and CBT techniques of cognitive reconstruction and behavioral training were incorporated throughout the entire intervention as technical tools. This intervention targeted towards negative emotions, irrational illness perception, and maladaptive coping strategies thereby helping working-age CRC patients to adapt to the sick role. The causal model of this study is presented in Fig. 1.

This study aims to develop a study protocol for a self-led VR-based CBT intervention to facilitate the sick role adaptation, modify irrational illness cognition, alleviate anxiety and depression, and improve QOL among working-age CRC patients and to evaluate its effectiveness.

## Hypotheses

We hypothesize that that working-age CRC patients in the VR-CBT group, in comparison with participants assigned to the attention control (AC) group, will report significantly better adaptation to the sick role, more rational illness cognition, lower levels of anxiety and depression and better QOL, at 1, 2 and 3-month after the intervention.

### Study design

A single center, parallel mixed-methods, assessor-blinded, randomized controlled trial will be conducted to investigate the effect of VR-based CBT on the sick role adaptation in working-age CRC patients with colorectal cancer. The protocol will adhere to the SPIRIT 2013 Statement guidelines for designing and reporting RCTs [36]. This study was registered on 21 June, 2023 in Chinese Clinical Trial Registry (No.: ChiCTR2300072699) at https://www.chictr.org.cn/.

### Sample/participants

The participants will be recruited from patients admitting to colorectal surgery ward with 70 beds at a tertiary-A cancer center in Guangzhou, Guangdong province, which is the largest integrated cancer center in the southern China.

Patients will be involved in the study if they: (1) are 18 ~ 59 years old, considering the retirement age can be no later than 60 in China, where people over this age are generally considered the elderly [37, 38]; (2) have been diagnosed with primary colon cancer or rectal cancer staging I-III in the past 3 months; (3) are informed diagnosis; (4) are able to comprehend spoken Mandarin and read Chinese; (5) possess and proficiently use a smartphone; and (6) consent to participate. Patients with (1) severe physical or mental health conditions, or (2) a history of psychotic illness or (3) severe motion sickness or vertigo in the past will be excluded.

### Sample size and recruitment

The primary outcome of the sick role adaptation in this study was used to calculate the effect size. Given the little published data on the effectiveness of VR-assisted CBT on the sick role adaptation among cancer patients, the sample size was calculated using PASS 15.0 based on a prior study in which a CBT intervention was delivered to elderly inpatients and yielded a s statistically significant improvement in the sick role adaptation. The mean score of the sick role dimension of the intervention group was (4.8 ± 1.2) and (6.6 ± 1.5) of the control groups. A sample size of 23 participants per group, assuming a more moderate effect size of 0.5, is predicted to provide the study with 95% power at a 1% level of significance to detect an effect size of at least 0.5 for the study's primary outcome of the sick role adaptation following the intervention. There will be a need for a total of 60 participants, with 30 in each arm, assuming a 20% attrition rate.

Eligible participants will be recruited through poster advertisements in wards and referrals from physicians. Then they will be approached by a research nurse who will distribute information sheets detailing the VR-CBT app and relevant consent forms. Prior to randomization, baseline data will be collected after receiving written consent. Data for follow-up will be collected at 1, 2 and 3-month after the completion of the 7-week sessions to examine the sustainability of the effects. The recruitment of participants will be completed by December 2023. Once recruited, patients will be randomly assigned to the VR-CBT group or AC group.

### Randomization and blinding

Participants will be assigned to the VR-CBT group or AC group using a stratified randomization, with an allocation ratio of 1:1, to ensure the equal distribution in colon cancer and rectum cancer patients throughout the recruitment period. A random sequence of grouping identifiers will be produced in advance using computer-generated random numbers by a researcher who does not participate in the trial and determine the eligibility of the subjects. According to the enrollment order and the associated group identifier in the previously created random sequence list, the group allocation for each participant will be distributed consecutively. Because of the nature of psychological interventions adopted in the study, it is difficult to conceal participants and the researchers who administer the intervention, researchers who will assess the outcomes will be blinded solely and not be notified of the group status of the participants.

## Intervention

### The attention control intervention

Threats to internal validity in RCTs will be reduced by the use of an AC group [36]. Participants assigned to the AC group will receive 7-week regular health consultations through WeChat as an attention control in addition to standard care by an experienced clinical nurse from the research setting. The nurse will contact participants in the AC group weekly from 1 week pre-surgery to 6 weeks post-surgery, to ask about recent health status using standardized questions such as ‘How do you feel today?’ ‘How is your appetite?’ and ‘How was your sleep last night’ and give general advice.

### The VR-CBT intervention

**Fig. 2:**
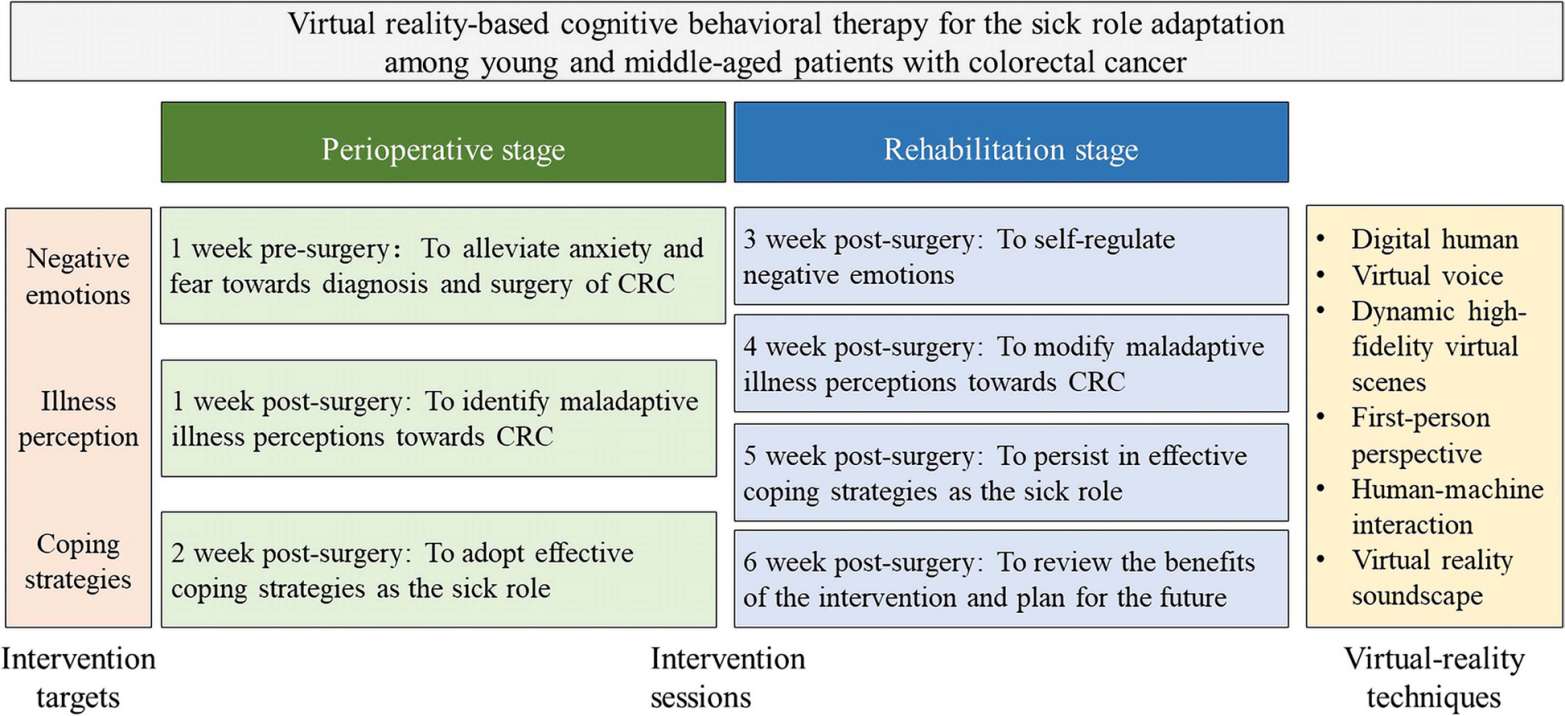
The overview of intervention targets, sessions in perioperative stage and rehabilitation stage, and adopted VR techniques

Participants in the VR-CBT group will be asked to complete a 7-sessions VR-CBT via a mobile app added onto standard care. We believe that this format will be more acceptable and available for working-age adults with CRC. The intervention is based on CSM targeted for negative emotions, irrational illness cognition, maladaptive coping strategies and supported by the use of CBT techniques during the intervention. According to psychological characteristics of patents in perioperative and rehabilitation stage, the weekly intervention will sequentially provide techniques of negative emotion adjustment, illness cognition reconstruction, and effective coping behaviors as the sick role at two different stages, from pre-surgery to 6 weeks post-surgery. Each session is estimated to take 40 min to complete. Patients will be allowed to repeat sessions at any time if they want within the intervention weeks. Figure 2 shows the overview of intervention targets, sessions in perioperative stage and rehabilitation stage, and adopted VR techniques.

**Fig. 3:**
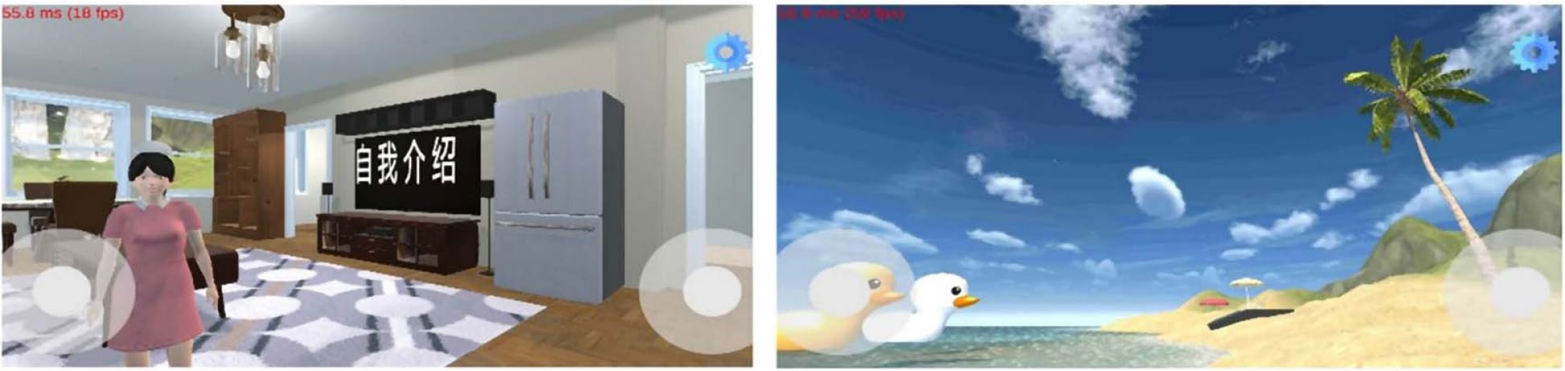
The visual representations of the app content

Based on Unity software (version 2018.3.11fl), we developed a self-led VR-CBT app that included comprehensive components of CBT in collaboration with School of Computer Science and Engineering, Sun Yat-sen University. The desktop VR application, classified as non-immersive in comparison to VR solutions using a head-mounted display, was adopted in this study. The avatars and structures constructing the virtual environment were built using Blender 2.83. The app has been registered in Copyright Protection Centre of China (Certificate No.11288804), and has been licensed by the Ministry of Industry and Information Technology of China (No. ICP 2022124889). The first phase of this app development involved an extensive, iterative process with numerous rounds of user testing. The app is designed with multimodal characteristics, including user-friendly interface, human–computer interaction and VR soundscape (therapeutic music, sound of waves, seagulls, footsteps, etc.) to strengthen immersion. Participants can interact in first-person perspective with the dynamic high-fidelity virtual environment by acting as a mouse pointer. Figure 3 shows the visual representations of the app content. Participants will be asked to install the app on their smartphones for free and instructed to use the app by the research nurse and provided with a user guide to introduce the functions in the app and provide solutions to problems, noting that the sessions should be completed in order and that each subsequent session will become available when the preceding one completed. Each participant will be assigned a unique key to login the app, while AC group will have no access to login to avoid contamination.

The VR-CBT app is consisted of three parts, including health education, providing CBT techniques, e.g., relaxation techniques practices, which are more applicable to immersive environment with virtual soundscape for patients to ‘escape from’ their real world, and summary with quiz of current day’s session to examine whether patients master the techniques. The intervener is a digital human in the app, in this study, a virtual nurse whose speech and body language were well-designed by the research team. The dynamic high-fidelity virtual scenarios in the app include indoor environment and sunshine beach to increase interest and adherence. The virtual nurse will teach patients cognitive behavioral strategies and assign homework with virtual voice in indoor environment, where patients will interact with virtual nurse. On the contrary, patients will practice breath relaxation and muscle relaxation techniques in sunshine beach environment with therapeutic music and seaside soundscape. Patients will submit their weekly homework in ‘Homework’ section the main interface. Table 1 provides the schedule of the VR-CBT intervention. The contents were designed and reviewed by clinical nurse managers and psychotherapists from Sun Yat-sen University Cancer Center and School of Nursing, Sun Yat-sen University, Guangzhou, Guangdong province, China.

**Table 1:** The schedule and details of the VR-CBT intervention

Stages	Sessions (Timepoint)	Themes	Contents	Assessments
Perioperative stage	1 (1 week pre-surgery)	What can I do with my anxiety and fear?	• Health education on diagnoses and treatments of CRC • Introduction to cognitive patterns of anxiety and fear as CRC patients • Breath relaxation technique with real-time assessment • Key points summary and quiz • Homework assignment: Patients record the use of breath relaxation technique	SSQ
	2 (1 week post-surgery)	Are my thoughts valid and useful?	• Health education on post-surgery early ambulation of CRC • Identification and evaluation of patients’ automatic thoughts towards CRC • Key points summary and quiz • Homework assignment: Patients record the automatic thoughts in the app	
	3 (2 weeks post-surgery)	Confrontation rather than avoidance	• Health education on enterostomy self-care or Kegel exercises • Effective behavioral strategies for adapting to the sick role • Appliance of behavioral experimental techniques to validate strategies • Key points summary and quiz • Homework assignment: Patients perform behavioral experiment and record results	
Rehabilitation stage	4 (3 weeks post-surgery)	My emotions are under my control	• Health education on physical activities of CRC survivors • Daily activities related to positive emotions • Muscle relaxation technique with real-time assessment • Key points summary and quiz • Homework assignment: Patients record the use of muscle relaxation technique	
	5 (4 weeks post-surgery)	Think it in a different way	• Health education on nutrition and dietary of CRC survivors • Psychoeducation of irrational illness and role beliefs • Key points summary and quiz • Homework assignment: Patients record the irrational and rational illness beliefs	
	6 (5 weeks post-surgery)	Actions speak louder than words	• Health education on health promotion behaviors of CRC survivors • Problem solving to the role maladaptation, including goal setting and time allocation • Key points summary and quiz • Homework assignment: Patients record behaviors that aim to adapt to the sick role	
	7 (6 weeks post-surgery)	Review and cope in the future	• Health education on follow-up visits of CRC survivors • Review the change of emotion, cognition, behaviors and CBT technique in the intervention • Gratitude to the participants and encouragement of coping in the future with CBT techniques	SSQ, semi-structured interviews

A master of Nursing Specialist who has undertaken the CBT training will monitor the adherence to the app protocol by tracking the number of sessions and homework that participants accessed in the backstage of app. Additionally, the intervention nurse will be responsible for checking the completion of patients submitted homework. More importantly, the intervention nurse will be tailored to address the issues patients encountered in completing homework.

The most common side effect associated with VR is simulator sickness, or VR sickness, cybersickness. It is a symptom similar to motion sickness that may manifest both during and after exposure to various VR environments, such as headaches, dizziness, and nausea during and after using VR devices [39, 40]. To reduce simulator sickness, we have paid extra attention to the various aspects of a VR system that contributed to users’ discomfort while designing the system. First, the app is based on desktop VR technique which is non-immersive and has the least potential for arising motion sickness. Second, the environment in VR-CBT app do not overly emphasize fast movement or rotation, and participants will have full control of their own motion in the VR environment. Third, to decrease the risk of dizziness, before entering the VR environment, the system will send prompt messages automatically to remind patients to avoid excessive movement or sudden head movements. Furthermore, we have increased and stabilized the frame rate (from 30 Frames Per Second to 60 Frames Per Second) to reduce the potential for dizziness, as the high display frame refresh rate is considered a crucial approach for alleviating the dizziness in VR applications [41]. In addition, the app improves the stability of VR scenario to reduce camera jitter or position changes that may cause dizziness.

Several strategies will be adopted to prevent attrition of study participants. Messages via WeChat will be sent weekly to keep in touch with patients after discharge. In addition, in the case of failure to reach participants, their main caregivers will be contacted. Incentives will be offered to all participants to expresses our gratitude when evaluating outcome variables at baseline, 1, 2 and 3-month post-intervention.

The intervention will be terminated if the participant declares he or she no longer wishes to participate, or is unable to continue the protocol treatment owing to adverse events. If the intervention proves to be effective, the VR-CBT app will be free available for participants enrolled in the AC group. Project management group meeting will be conducted weekly and blind information on the study.

## Outcome measures

### Primary outcome

The sick role adaptation will be measured using the Chinese version of Illness Behavior Questionnaire (IBQ). IBQ has been used to predict response to illness and treatment [42]. The Chinese version of IBQ could be divided into 3 dimensions, namely negative emotions, behavioral responses and the sick role [43]. The Chinese version of the IBQ has demonstrated acceptable internal consistency, with Cronbach’s α coefficient 0.680–0.765 [43].

### Secondary outcomes

The negative emotions will be assessed using the Hospital Anxiety and Depression Scale (HADS). It is a self-report questionnaire comprising subscales for anxiety and depression. A total of 14 items included in the scale, each of which is scored on a Likert 4-point scale from 0 to 3, with a total score of 0 to 7 for being asymptomatic, 8 to 10 for mild anxiety or depression symptoms, and 11 to 21 for significant anxiety or depression symptoms [44]. The Chinese version of HADS revealed a Cronbach's alpha coefficient of 0.88 [45].

The illness cognitions will be assessed using the Brief Illness Perception Questionnaire (BIPQ). The BIPQ consisted of nine items that examine the patient’s cognitive and emotional responses to illness [46]. The items address perceived consequences, timeline, personal control, treatment control, identity, concerns, comprehension, and emotional response. The final item is an open-ended response question that assesses causes of the illness. The Chinese version of BIPQ has been widely used as a screening tool for assessing illness perceptions in China, with the Cronbach's α and split-half reliability of BIPQ was 0.77 and 0.81 respectively [47].

The QOL will be assessed using the European Organization for Research and Treatment of Cancer Quality of Life Questionnaire-Core 30 (EORTC QLQ-C30) [48]. It includes five functional scales (physical, role, emotional, cognitive and social), three symptom scales (pain, fatigue and nausea/vomiting), one global health status scale and six single items of symptoms (e.g., insomnia). For both the overall scale and each scale, the raw scores are transformed into standard scores in the range of 0 to100. Higher scores on the scale indicate better QOL. The Chinese version of EORTC QLQ-C30 [49] has demonstrated satisfactory internal consistency reliability and structural validity.

### Other measurements

The socio-demographic and clinical characteristics of the participants will be collected, such as sex, age, educational level, marital status, household income per month, tumor location, stage and treatment modality.

Furthermore, semi-structured interviews will be employed to explore and gain insights into participants’ experiences and perceptions of the VR program until data saturation. The post-intervention interviews will be carried out following the completion of the final scheduled session and audio recorded after obtaining informed consent from the participants. Open-ended, probing questions will be used to guide the interview (Table 2). Content analysis approach will be applied to analyze the qualitative data.

**Table 2:** Topic guide for post-intervention interview

1. When do you usually use the app? Why? 2. How do you feel about VR program? 3. What impressed you most in the VR program? 4. How the VR program help you adapt to the sick role and cope with CRC? 5. Have you experience any discomfort or difficulties during the usage of the VR program? Please tell me details. 6. What are your thoughts on the homework? 7. What advice would you give us on the VR program? 8. If there is a chance, would you recommend the VR program to other patients? Why?	

In addition, login counts and time, length of usage, time spent of each session, quiz results and results of human–computer interaction will be collected via backend monitoring data to evaluate the completion of the intervention. On the other hand, the privacy issues will also be seriously taken into account. First, the app will not collect the personal information that may recognize the patient identity such as name, age, residence and diagnosis. Second, the user data will be stored in password-controlled, restricted-access servers in the university to protect user information from loss, misuse, unauthorized access or disclosure, alteration, or destruction. Third, the user behavior data will only be used for statistical analysis and supervised by the Institutional Review Board of School of Nursing, Sun Yat-sen University.

### Side-effect measurement

The most widely used measurement of VR related side-effect was the Simulator Sickness Questionnaire (SSQ), developed by Kennedy et al. [50]. The SSQ consists of 16 items with answers ranging from 0 to 3 based on the severity of the participant's symptoms. The SSQ includes three subscales: nausea, oculomotor and disorientation. The overall score serves as an indicator of total severity of the simulator sickness, with a higher the SSQ score indicating more severe simulator sickness [39]. The Chinese version of SSQ showed acceptable structure validity and reliability [51].

### Data collection procedure

**Fig. 4:**
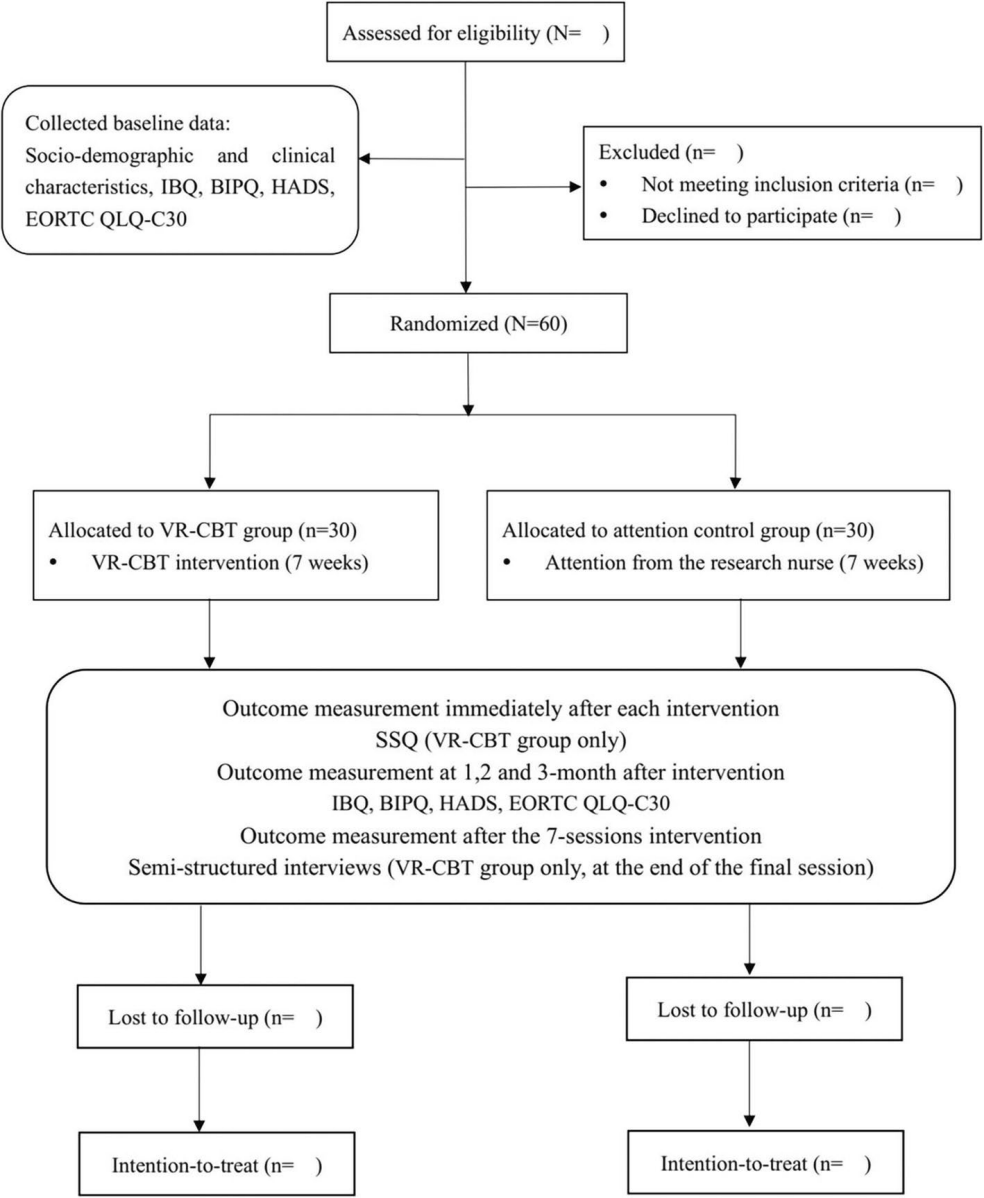
The Consolidated Standards of Reporting Trials flowchart

The Consolidated Standards of Reporting Trials flowchart is presented in Fig. 4. The research nurse who is responsible for participant recruitment and random group assignment will carry out baseline evaluations. Each participant will be required to complete the Chinese versions of the IBQ, HADS, BIPQ, EORTC-QLQ C30 and the socio-demographic questionnaires at colorectal oncology ward. Data collection will be carried out through electronic questionnaires distributed to discharged participants by a research nurse who will remain blinded to the group allocation of the participants. Immediately following the program's final session, another research nurse will conduct semi-structured interviews with the participants in VR-CBT group. If the research nurse fails to reach some participants, their main caregivers will be contacted and remind the participants to complete the follow-up.

All data will be stored on a secure and password-protected database, which will be accessible to the principal researchers involved in the study for the purpose of analysis and reporting. Review of data collected by other researchers is not permitted. Original documentation and signed informed consent forms will be kept in lockable file cabinets.

### Data analysis

Data will be analyzed using IBM SPSS 26.0 (IBM Corp.). Demographic data will be described and presented with appropriate statistics, including frequency distributions, means with standard deviations, medians with interquartile ranges. In the case of skewed variables, appropriate transformations will be applied prior to their inclusion in the inferential analysis. The outcome analysis will adhere to the intention-to-treat principle. The homogeneity of baseline characteristics between the two groups will be assessed using appropriate statistical tests including independent *t*- test, chi- squared test or Fisher's exact test. The study will apply the generalized estimating equations model adjusting for specific covariates (sex, age, monthly income per capita, family history of cancer, stage of cancer, and time), to compare the changes in each outcome variables between the two groups across different time points. Multiple imputation will be adopted to handle missing data. All statistical tests employed in this study will be two-tailed, with a significance level set at 0.05.

### Validity and reliability

This study employs a randomized controlled trial design to investigate the effects of a VR-based CBT intervention with a well-represented sample, by using validated instruments for data collection and rigorous statistical analysis. Additionally, blinding the outcome assessors to the group allocation status of participants can effectively minimize bias and enhance the generalizability of the findings to the broader target population. These measures ensure that the assessment of outcomes remains objective and unbiased, as the assessors are unaware of which participants belong to which group. By eliminating potential bias, the study findings can be applied more reliably to a wider population, increasing the external validity of the results. Furthermore, the intervention proposal has been reviewed and modified by relevant experts to address the uncertainty or incomplete information of the study protocol.

## Discussion

To the best of our understanding, this will be the first randomized controlled trial of a self-led VR-based CBT intervention for working-age CRC patients in China. If the intervention shows promising results and favorable user experience, it will generate new insights to the advancement of theoretical and practical knowledge of CBT interventions which focus on irrational illness belief, negative emotions and ineffective coping styles and aim to promote psychosocial adaptation. Furthermore, it will add to the evidence that self-led VR-based CBT has potential to serve as an accessible and feasible alternative to traditional high-cost treatment and applies in cancer survivorship care settings to improve the quality of patient-centered care and outcomes of younger cancer survivors.

## Limitations

This study is subject to some possible limitations that should be acknowledged. First, blinding of participants will not be achievable due to the nature of the intervention. Performance bias could be introduced as a result. Nevertheless, to minimize bias in evaluating the effects of the intervention, outcome assessors will be blinded. Second, though the app has been based on desktop VR technology, it is still possible for participants to experience simulation sickness. To address this potential adverse effect, we will remind the participants of the recommended usage time and break time in the operation manual to reduce the risk of simulation sickness.

## Conclusions

This study will add to the existing body of knowledge and advance research in the field on the effectiveness of a self-led, VR-based CBT intervention targeted for psychosocial outcomes on cancer populations. If the VR-based CBT intervention demonstrates its effectiveness in improving sick role adaptation in working-age adults with CRC, the next step will be to popularize this type of intervention on negative emotions, illness perception and coping styles to address their psychosocial challenges and age-tailored concerns.

## Data Availability

This paper is a study protocol with not direct data generated. Nonetheless, information on project development can be addressed through correspondence author ( zhmfen@mail.sysu.edu.cn ).

## Abbreviations

CRC: Colorectal cancer
CBT: Cognitive behavioral therapy
QOL: Quality of life
CSM: Common Sense Model of Self-regulation
VR: Virtual reality
VR-CBT: Virtual reality-based cognitive behavioral therapy
AC: Attention control
IBQ: Illness behavior questionnaire
HADS: Hospital Anxiety and Depression Scale
BIPQ: Brief Illness Perception Questionnaire
EORTC QLQ-C30: European Organization for Research and Treatment of Cancer Quality of Life Questionnaire-Core 30
SSQ: Simulator Sickness Questionnaire
